# Barriers to and Disparities in Access to Health Care Among Adults Aged ≥18 Years with Epilepsy — United States, 2015 and 2017

**DOI:** 10.15585/mmwr.mm7121a1

**Published:** 2022-05-27

**Authors:** Niu Tian, Rosemarie Kobau, Matthew M. Zack, Kurt J. Greenlund

**Affiliations:** 1 Division of Population Health, National Center for Chronic Disease Prevention and Health Promotion, CDC.

Approximately 3 million U.S. adults have active epilepsy (i.e., self-reported doctor-diagnosed history of epilepsy and currently taking epilepsy medication or have had at least one seizure in the past year, or both) (*1*). One of the most common brain disorders, epilepsy poses a number of challenges for people living with this condition because its treatment can be complex, daily management might be inadequate to achieve seizure control, it limits social participation, and epilepsy is associated with early mortality.^†^ Previous studies indicate that persons with epilepsy are more likely to experience barriers or delays in receipt of certain types of care, including epilepsy specialty care, and that these delays are often associated with individual factors (e.g., seizure type) or social determinants of health (e.g., household income or provider availability) (*2*–*4*). To obtain updated estimates of access to health care among U.S. adults aged ≥18 years by epilepsy status, CDC analyzed pooled data from the 2015 and 2017 National Health Interview Survey (NHIS), the most recent years with available epilepsy data. Age-adjusted analyses comparing adults with active epilepsy or inactive epilepsy (i.e., self-reported doctor-diagnosed epilepsy but not currently taking medication for epilepsy and have had no seizure in the past year) with adults without epilepsy indicated that adults with active or inactive epilepsy were more likely to have Medicaid or other public insurance coverage and to report an inability to afford prescription medicine, specialty care, or vision or dental care. Adults with active or inactive epilepsy were more likely to take less medication than prescribed to save money, to be in families having problems paying medical bills, and to report delaying care because of insufficient transportation. Enhancing linkages between clinical and community programs and services by public health practitioners and epilepsy health and social service providers can address gaps in access to health care.

NHIS is an annual, nationally representative household survey of the U.S. civilian, noninstitutionalized population.^§^ Supplementary questions on epilepsy were added to the 2015 and 2017 Sample Adult Core component of NHIS, which includes one randomly selected adult aged ≥18 years from each randomly selected household. Adult respondents answered three questions about epilepsy to self-identify as a person with active, inactive, or no epilepsy (epilepsy status).^¶^ These case-ascertainment questions have been validated for use in community surveillance (*5*). Information about access to health care and income was collected in the NHIS Sample Adult, Person, and Imputed Income files. In 2015 and 2017, a total of 33,672 adults (final response rate = 55.2%) and 26,742 adults (final response rate = 53.0%), respectively, responded to the survey.** CDC pooled 2015 and 2017 data (combined response rate = 54.1%) to increase the reliability of estimates.

Estimates were weighted and age-standardized to the 2000 U.S. Census Bureau projected adult population using three age groups: 18–44, 45–64, and ≥65 years.^††^ Age-standardized prevalences of selected access-to-care indicators^§§^ were compared between adults with active epilepsy and no epilepsy and between those with inactive epilepsy and no epilepsy. Age-standardized percentages of adults with active, inactive, and no epilepsy who were in families having problems paying medical bills in the past year were calculated by selected sociodemographic characteristics. Analyses were conducted with SAS-callable SUDAAN (version 9.4; SAS Institute) to account for the respondent sampling weights and NHIS complex sample design. All reported differences are statistically significant (p<0.05 by two-tailed t-tests). After excluding respondents with missing information on epilepsy history (i.e., respondents who refused to respond or responded “don’t know” to the question “Have you ever been told by a doctor or other health professional that you have a seizure disorder or epilepsy?”), the final analytical sample included 60,281 (99.0%) respondents. This activity was reviewed by CDC and was conducted consistent with applicable federal law and CDC policy.^¶¶^

During 2015 and 2017, adults were less likely to be uninsured if they had active (6.5%) epilepsy compared with those without epilepsy (11.0%) (Table 1). Adults with active or inactive epilepsy were less likely to have private insurance (39.3% and 53.9%, respectively) and more likely to have Medicaid or other public health insurance coverage (44.4% and 27.3%, respectively) than were those without epilepsy (64.9% [private] and 15.6% [Medicaid or other public health insurance]). More adults with active epilepsy than without epilepsy had trouble finding a doctor or other health care provider (5.4% versus 3.1%). More adults with active epilepsy (6.9%) or inactive epilepsy (4.9%) than without epilepsy (1.7%) reported delayed care because of lack of transportation. A greater percentage of adults with active or inactive epilepsy had difficulty affording a specialist (7.5% and 7.3%, respectively) than did those without epilepsy (4.1%); a similar pattern was observed for affording mental health care.

**TABLE 1:** Crude and age-standardized prevalences* of indicators of limitations in access to care among adults aged ≥18 years, by epilepsy status — National Health Interview Survey, United States, 2015 and 2017

Characteristic	% (95% CI)					
	Active epilepsy^†^ (n = 735)		Inactive epilepsy^§^ (n = 456)		No epilepsy^¶^ (n = 59,090)	
	Crude	Age-standardized	Crude	Age-standardized	Crude	Age-standardized
**Current insurance type**						
Private	39.2 (34.4–44.2)	39.3 (34.4–44.4)**	55.6 (49.7–61.4)	53.9 (48.0–59.6)**	64.4 (63.7–65.1)	64.9 (64.2–65.6)
Medicaid/Other public^††^	46.6 (41.7–51.5)	44.4 (39.8–49.1)**	27.9 (22.8–33.7)	27.3 (22.2–33.0)**	15.4 (14.9–15.8)	15.6 (15.1–16.1)
Medicare	8.0 (6.1–10.5)	9.8 (8.2–11.7)	5.5 (3.7–8.1)	8.0 (6.0–10.5)	10.0 (9.6–10.3)	8.6 (8.4–8.8)
Uninsured	6.3 (4.2–9.3)	6.5 (4.4–9.7)**	10.9 (10.6–15.4)	10.8 (7.6–15.3)	10.3 (9.9–10.7)	11.0 (10.5–11.4)
**Reasons for not seeking care, paying medical bills, or obtaining prescriptions when needed during the last 12 months**						
Lack of transportation	7.11 (5.4–9.3)	6.9 (5.2–9.1)**	5.0 (3.3–7.6)	4.9 (3.2–7.4)**	1.8 (1.6–1.9)	1.7 (1.6–1.9)
Trouble finding provider who would see them	5.7 (3.9–8.0)	5.4 (3.7–7.8)**	4.5 (2.7–7.4)	4.4 (2.7–7.2)	3.1 (2.9–3.3)	3.1 (2.9–3.3)
Could not afford to see a specialist	7.6 (5.4–10.7)	7.5 (5.3–10.6)**	7.6 (5.2–10.9)	7.3 (5.0–10.4)**	4.1 (3.9–4.4)	4.1 (3.9–4.4)
Could not afford mental health care or counseling	4.4 (2.9–6.8)	4.4 (2.8–6.8)**	5.6 (3.2–9.4)	5.4 (3.1–9.3)**	1.9 (1.8–2.1)	2.0 (1.9–2.2)
Last ED visit because didn’t have another place to go^§§^	36.4 (29.8–43.6)	38.4 (31.2–46.1)	44.7 (34.8–55.0)	43.7 (34.4–53.5)	39.6 (38.2–40.9)	40.3 (38.9–41.8)
Problems paying medical bills^¶¶^	28.4 (24.0–33.2)	27.9 (23.5–32.8)**	27.8 (22.9–33.2)	27.6 (22.7–33.1)**	13.8 (13.4–14.3)	14.0 (13.6–14.5)
Could not afford prescription medicines	13.6 (10.7–17.2)	13.2 (10.3–16.7)**	12.6 (9.6–16.3)	12.4 (9.3–16.2)**	6.1 (5.8–6.3)	6.1 (5.8–6.3)
Skipped medication doses to save money	9.7 (7.1–13.2)	9.3 (6.7–12.7)**	13.1 (9.4–18.0)	12.9 (8.9–18.2)**	5.9 (5.6–6.2)	6.1 (5.8–6.5)
Took less medicine to save money	10.5 (7.8–14.0)	10.8 (8.1–14.2)**	12.2 (8.6–16.9)	11.6 (7.9–16.6)**	6.2 (5.8–6.5)	6.4 (6.1–6.8)
Delayed filling prescription to save money	12.2 (9.4–15.6)	12.2 (9.4–15.7)**	15.4 (11.5–20.5)	14.9 (10.8–20.2)**	7.7 (7.4–8.1)	8.3 (7.9–8.7)
Could not afford dental care	20.9 (17.4–25.0)	20.3 (16.8–24.3)**	19.5 (15.4–24.5)	19.3 (15.1–24.2)**	10.7 (10.3–11.0)	10.7 (10.3–11.1)
Could not afford eyeglasses	12.9 (10.1–16.3)	12.5 (9.7-–5.9)**	10.2 (7.3–14.0)	9.6 (6.0–13.4)	6.0 (5.7–6.3)	5.9 (5.6–6.3)

**Abbreviation**: ED = emergency department.

* The percentage estimates are weighted. Estimates are age-standardized to the 2000 U.S. Census Bureau projected population, aged ≥18 years, using three age groups: 18–34, 35–64, and ≥65 years.

^†^ Active epilepsy was defined as adults who answered that a doctor or health professional had ever told them they had a seizure disorder or epilepsy and also reported taking medication, having had one or more seizures in the past year, or both.

^§^ Inactive epilepsy was defined as adults who reported a history of epilepsy but were not taking medication for epilepsy and had not had a seizure in the past year.

^¶^ No epilepsy was defined as adults who answered no history of ever having been diagnosed with epilepsy or seizure disorder by a doctor or health professional.

** A t-test was conducted to compare the prevalence estimates between adults with active epilepsy and without epilepsy and between adults with inactive epilepsy and without epilepsy in the same category of indicator of access to care at the statistical significance level of 0.05 (p<0.05 by two-tailed t-tests).

^††^ Other public included state-sponsored or state and federal jointly sponsored children’s health insurance program and any type of military coverage with or without Medicare or other government programs.

^§§^ Among adults with at least one ED visit in the past year.

^¶¶^ Problems paying bills was defined as answering “yes” to any of the following questions: “Did you/anyone in the family have problems paying or were unable to pay any medical bills in the past 12 months?” (this could include bills for doctors, dentists, hospitals, therapists, medication, equipment, nursing home, or home care) or “Do you/does anyone in your family currently have any medical bills that you are unable to pay at all?”

Adults with active or inactive epilepsy were more likely to report an inability to afford prescription medicine (13.2% and 12.4%), skipping medication doses to save money (9.3% and 12.9%), delaying obtaining refills (12.2% and 14.9%), taking less than the prescribed dosages of medicine to save money (10.8% and 11.6%), and being unable to afford dental care (20.3% and 19.3%) compared with those without epilepsy (6.1%, 6.1%, 8.3%, 6.4%, and 10.7%, respectively). Adults with active epilepsy were more likely to report an inability to afford eyeglasses (12.5%) than were those without epilepsy (5.9%).

Adults with active or inactive epilepsy were overall significantly more likely to be in families having problems paying their medical bills (27.9% and 27.6%, respectively) than were adults without epilepsy (14%) (Table 2). Selected subgroups of adults with active or inactive epilepsy (e.g., those aged <65 years and in a family earning <200% of the federal poverty status) were also more likely to be in families having problems paying medical bills.

**TABLE 2:** Numbers and age-standardized percentages* of living in a family having problems paying medical bills^†^ in the past year among adults aged ≥18 years, by epilepsy status — National Health Interview Survey, United States, 2015 and 2017

Characteristic	Active epilepsy^§^ (n = 735)		Inactive epilepsy^¶^ (n = 456)		No epilepsy** (n = 59,090)	
	No./Total no.^††^	Age-standardized % (95% CI)	No./Total no.^††^	Age-standardized % (95% CI)	No./Total no.^††^	Age-standardized % (95% CI)
**Total**	**202/735**	**27.9 (23.5–32.8)^§§^**	**112/456**	**27.6 (22.7–33.1)^§§^**	**7,768/59,090**	**14.0 (13.6–14.5)**
**Age group, yrs**						
18–44	85/284	30.9 (24.1–38.6)^§§^	47/187	28.7 (21.1–37.8)^§§^	3,535/23,846	15.4 (14.7–16.0)
45–64	97/298	30.5 (24.4–37.3)^§§^	50/178	27.8 (20.7–36.1)^§§^	3,083/19,818	15.1 (14.4–15.9)
≥65	20/153	14.1 (7.2–26.0)^¶¶^	15/91	23.7 (14.3–36.5)^§§^	1,150/15,426	8.0 (7.4–8.6)
**Sex**						
Men	81/333	24.5 (18.2–32.2)^§§^	46/166	31.5 (23.0–41.3)^§§^	3,098/26,606	12.61 (12.0–13.2)
Women	121/402	31.4 (25.7–37.8)^§§^	66/290	25.5 (19.6–32.4)^§§^	4,670/32,484	15.4 (14.8–16.0)
**Race/Ethnicity**						
White, non-Hispanic	129/517	25.6 (20.5–31.6)^§§^	78/330	26.0 (20.5–32.4)^§§^	4,477/38,553	12.7 (12.2–13.2)
Black, non-Hispanic	31/99	34.3 (23.4–47.1)^§§^	15/ 58	42.5 (26.9–59.8)^§§^	1,391/7,131	20.3 (19.0–21.8)
Hispanic	23/71	34.6 (21.9–50.0)^§§^	15/42	40.7 (26.3–56.9)^§§^	1,436/8,705	16.9 (15.8–18.1)
Other***	19/48	36.7 (20.9–55.9)^§§^	—^†††^	—^†††^	464/4,701	10.0 (8.9–11.3)
**Poverty status^§§§^**						
<100% (of FPL)	77/227	33.9 (26.6–42.0)^§§^	32/107	37.7 (26.4–50.4)^§§^	1,646/8,807	20.5 (19.2–21.9)
≥100% to <200%	70/196	43.4 (33.3–54.0)^§§^	37/107	40.3 (29.9–51.7)^§§^	2,410/11,433	23.5 (22.4–24.6)
≥200% to <300%	32/102	36.0 (23.7–50.6)^§§^	21/68	32.6 (18.9–50.2)	1,571/9,697	19.0 (17.8–20.2)
≥300% to <400%	14/80	17.9 (10.3–29.3)	8/45	24.1 (11.0–45.0)^¶¶^	912/7,437	14.3 (13.1–15.7)
≥400%	9/130	5.1 (2.3–10.7)^¶¶^	14/129	15.2 (8.7–25.2)^§§^	1,229/21,715	6.3 (5.8–6.8)
**Education level**						
Less than HS graduate	40/152	30.7 (22.3–40.7)^§§^	20/71	30.9 (19.8–44.8)	1,321/7,440	21.1 (19.6–22.6)
HS graduate or equivalent	53/217	26.2 (18.4–35.8)^§§^	33/119	32.2 (22.1–44.3)^§§^	2,151/14,423	16.8 (15.9–17.8)
Some college or more	106/356	26.1 (20.3–32.8)^§§^	58/263	24.0 (18.0–31.1)^§§^	4,270/37,015	11.7 (11.3–12.2)
**Current employment**						
Yes	47/192	18.7 (12.9–26.2)	50/209	26.8 (19.6–35.6)^§§^	4,446/34,524	12.6 (12.0–13.1)
No	155/543	31.5 (25.8–37.7)^§§^	62/247	28.3 (21.4–36.4)^§§^	3,320/24,543	17.8 (16.9–18.7)
**Marital status**						
Married/Living with partner	68/268	24.9 (18.7–32.2)^§§^	44/188	26.1 (19.2–34.4)^§§^	3,706/29,705	13.2 (12.6–13.7)
Widowed/Divorced/ Separated	76/240	33.7 (23.0–45.2)^§§^	44/145	38.1 (26.0–52.0)^§§^	2,340/15,911	19.4 (18.0–20.7)
Never married	57/226	26.0 (19.0–34.7)^§§^	24/123	24.5 (15.3–36.7)^§§^	1,709/13,357	13.5 (12.6–14.5)
**Region**						
Northeast	20/121	12.9 (6.0–25.6)^¶¶^	12/58	22.3 (11.0–40.2)^¶¶^	1,092/9,727	11.4 (10.4–12.3)
Midwest	36/161	26.1 (16.2–39.2)	32/121	29.4 (20.3–40.5)^§§^	1,724/13,137	15.1 (14.2–16.0)
South	97/ 287	36.3 (29.4–43.8)^§§^	49/163	36.3 (27.5–46.1)^§§^	3,251/21,005	16.6 (15.8–17.4)
West	49/166	24.1 (16.4–33.9)^§§^	19/114	17.2 (10.0–28.1)	1,701/15,221	11.1 (10.3–12.1)

**Abbreviations**: FPL = federal poverty level; HS = high school.

* The percentage estimates are weighted. Age-standardized to the 2000 U.S. Census Bureau projected population, aged ≥18 years, using three age groups: 18–44, 45–64, and ≥65 years. Estimates for age groups are not age-standardized (i.e., presented as crude percentages).

**^†^** Problem paying bills was defined as answering “yes” to any of the following questions: “Did you/anyone in the family have problems paying or were unable to pay any medical bills in the past 12 months?” (this could include bills for doctors, dentists, hospitals, therapists, medication, equipment, nursing home, or home care); or “Do you/does anyone in your family currently have any medical bills that you are unable to pay at all?”

^§^ Active epilepsy was defined as having a diagnosis of epilepsy and either taking medication, having had one or more seizures in the past year, or both.

^¶^ Inactive epilepsy was defined as adults who reported a history of epilepsy but were not taking medication for epilepsy and had not had a seizure in the past year.

** No epilepsy was defined as adults who answered no history of ever having been diagnosed with epilepsy or seizure disorder by a doctor or health professional.

^††^ “Total number” represents unweighted numbers of those with active epilepsy, inactive epilepsy, or no epilepsy (denominator); “number” represents unweighted numbers of those living in a family having problems paying bills among those with active epilepsy, inactive epilepsy, or no epilepsy (numerator). Some of the categories do not sum to the total (e.g., education level or marital status) and categories might not sum to the sample total because of missing responses.

^§§^ A t-test was conducted to compare the prevalence estimates between adults with active epilepsy and without epilepsy and between adults with inactive epilepsy and without epilepsy in the same category of characteristics at the statistical significance level of 0.05 (p<0.05 by two-tailed t-tests).

**^¶¶^** Estimate is unreliable because the relative SE was >30% but <50%. Results should be interpreted with caution.

*** The Other race and ethnicity category includes other non-Hispanic groups (American Indian or Alaskan Native, Asian, multiple race, and race group not releasable).

^†††^ Number and estimate were suppressed because denominator was <30 or relative SE was >50%.

^§§§^ Poverty status was defined as the ratio of family income to federal poverty level. Estimates were calculated from the National Health Interview Survey income data file.

## Discussion

Healthy People 2030 objectives include reducing the proportion of persons who cannot obtain needed medical care and reducing the proportion of persons who cannot obtain necessary prescription medicines (*6*). Persons with epilepsy need access to medical care for both epilepsy (e.g., access to antiseizure medication and neurologists) and nonepilepsy-related medical care (e.g., access to dental and vision care) to prevent comorbidity, worsening health status, and early mortality (*7*). The findings in this study indicate a broad range of barriers for both epilepsy- and nonepilepsy-related medical care that might complicate epilepsy management and increase comorbidities, hospitalizations, disability, and health care costs for those living with the disorder as well as for those with a history of epilepsy.

Consistent with a previous study based on 2010 and 2013 NHIS data, adults with active epilepsy were more likely to be insured with Medicaid or other public insurance coverage than were those without epilepsy (*3*). Medicare coverage might afford some protection against problems paying medical bills for adults with active epilepsy aged ≥65 years compared with younger adults with epilepsy who are not eligible for Medicare. However, a 2013 study found that fewer California adults with active epilepsy who had Medicare or Medicaid obtained specialized epilepsy care compared with adults with private insurance (*4*). Medicaid expansion reduced cost-related barriers to care and was associated with improvements in selected health outcomes among low-income adults with chronic disease (*8*). Medicaid coverage for those who qualify includes mandatory benefits (e.g., outpatient hospital services) and optional benefits (e.g., prescription drugs, non-emergency medical transportation, dental care, and optometry care), which vary by state. The extent to which services are covered by Medicaid might facilitate or limit access to these services for adults with active epilepsy.***

Other individual-level factors such as sex, presence of comorbidities, or health literacy and contextual factors that constitute social determinants of health (e.g., reliable transportation, provider availability or cultural competency, and lower rates of public insurance reimbursement) might also influence epilepsy care and outcomes (*4*,*9*). Although all racial and ethnic groups with active epilepsy were more likely to report having problems paying medical bills than their counterparts without epilepsy, assessing differences in problems paying medical bills within racial and ethnic groups requires more study with larger samples. Additional studies are warranted to examine health inequities associated with race and ethnicity and social determinants of health by epilepsy status. Finally, an epilepsy diagnosis earlier in life has been reported to alter neurodevelopment and might limit opportunities later in life (*10*). More studies are needed to examine the challenges faced by adults with inactive epilepsy.

The findings in this report are subject to at least five limitations. First, because NHIS is cross-sectional, causal inferences related to the association between health care access barriers and epilepsy status cannot be made. Second, estimates are based on self-reported data and might be subject to reporting bias. Third, because adults aged ≥65 years without private insurance can have both Medicare and Medicaid coverage, the percentages of adults with Medicare might include some who are eligible for Medicaid (i.e., “dually eligible”), potentially leading to an underestimate of the overall percentages with Medicaid coverage by epilepsy status. Fourth, it is not known whether problems paying medical bills reported by a respondent with active or inactive epilepsy are related to the respondent’s own medical bills or those of other family members.^†††^ Finally, because more recent data are not available, the findings from this analysis might not represent associations of these factors beyond 2017. The ongoing efforts for data modernization and enhanced linkages with electronic health records might improve availability of more data to guide public health action.

Public health practitioners and epilepsy health and social service providers can raise awareness of the CDC-supported Epilepsy Foundation Epilepsy and Seizures 24/7 Helpline, which has trained English- and Spanish-speaking information specialists available 24 hours a day by phone and email to refer persons to local community-based programs such as medication assistance programs, transportation services, and other resources.^§§§^ The Epilepsy Foundation also provides information to assist patients in finding epilepsy centers and specialists nationwide.^¶¶¶^ Addressing disparities in access to care necessitates a comprehensive approach that accounts for social determinants of health (*6*,*9*) and intervenes to reduce treatment gaps. Public health practitioners and epilepsy health and social service providers can enhance linkages between clinical and community programs and services to address gaps in access to health care.

**Summary**

**What is already known about this topic?**

Adults with epilepsy are more likely to experience barriers to accessing health care than are adults without epilepsy.

**What is added by this report?**

In 2015 and 2017, compared with U.S. adults without epilepsy, adults with active or inactive epilepsy were more likely to report an inability to afford prescription medicine, specialty care, or other types of care, had trouble finding a doctor, delayed care because of transportation barriers, or were in families having problems paying medical bills.

**What are the implications for public health practice?**

Public health practitioners and epilepsy health and social service providers can enhance linkages between clinical and community programs and services to address gaps in access to health care.

## Footnotes

* Deceased.

† https://www.nap.edu/read/13379/chapter/3#25

§
https://www.cdc.gov/nchs/nhis/index.htm

¶ 1) “Have you ever been told by a doctor or other health professional that you have a seizure disorder or epilepsy?” 2) “Are you currently taking any medicine to control your seizure disorder or epilepsy?” 3) “Think back to last year about the same time. About how many seizures of any type have you had in the past year?” Active epilepsy was defined as having a diagnosis of epilepsy and either taking medication, having had one or more seizures in the past year, or both. Inactive epilepsy was defined as adults who reported a history of epilepsy but were not taking medication for epilepsy and had not had a seizure in the past year. Adults with no epilepsy were those who answered no history of ever having received a diagnosis of epilepsy or seizure disorder by a doctor or health professional.

** https://nhis.ipums.org/nhis/resources/srvydesc2015.pdf; https://ftp.cdc.gov/pub/Health_Statistics/NCHS/Dataset_Documentation/NHIS/2017/srvydesc.pdf

††
https://www.cdc.gov/nchs/data/statnt/statnt20.pdf

§§ NHIS Person file (Family questionnaire) access-to-care indicators include insurance type; respondent or a family member having problems paying medical bills; and having medical bills that cannot be paid at all. Sample Adult file access-to-care indicators include no transportation to get to a doctor’s office in the past 12 months; trouble finding a doctor/provider in the past 12 months; couldn't afford seeing a specialist in the past year; couldn’t afford mental health care or counseling in the past 12 months; had an emergency department visit because of not having another place to go (among adults who had an emergency department visit in the past year); couldn’t afford dental care or eyeglasses in the past 12 months; couldn’t afford prescription medicine in the past 12 months; and skipped medication doses/took less/delayed filling prescription to save money in the past 12 months.

¶¶ 45 C.F.R. part 46.102(l)(2), 21 C.F.R. part 56; 42 U.S.C. Sect. 241(d); 5 U.S.C. Sect. 552a; 44 U.S.C. Sect. 3501 et seq.

***
https://www.medicaid.gov/medicaid/benefits/index.html

†††
https://www.cdc.gov/nchs/data/nhis/earlyrelease/probs_paying_medical_bills_jan_2011_jun_2017.pdf

§§§
https://www.epilepsy.com/connect/247-helpline

¶¶¶
https://www.epilepsy.com/connect/find-epilepsy-specialist
